# Antimicrobial and Antioxidant Edible Films and Coatings in the Shelf-Life Improvement of Chicken Meat

**DOI:** 10.3390/foods12122308

**Published:** 2023-06-07

**Authors:** Márcio Moura-Alves, Alexandra Esteves, Maria Ciríaco, José A. Silva, Cristina Saraiva

**Affiliations:** 1 CECAV—Animal and Veterinary Research Centre, University of Trás-os-Montes e Alto Douro (UTAD), 5000801 Vila Real, Portugal; alexe@utad.pt (A.E.); mariaciriaco@utad.pt (M.C.); jasilva@utad.pt (J.A.S.); crisarai@utad.pt (C.S.); 2 AL4AnimalS—Associate Laboratory for Animal and Veterinary Sciences, 5000801 Vila Real, Portugal

**Keywords:** active packaging, edible films, edible coatings, antimicrobial properties, antioxidant properties, shelf-life

## Abstract

Meat deterioration during processing, distribution, and display can compromise the quality and safety of products, causing several undesirable changes and decreasing products’ shelf-life, which has a negative impact on the industry and consumers. In recent years, studies have been carried out using decontamination techniques and new packaging methodologies to overcome deterioration problems, increase sustainability, and reduce waste. Edible films and coatings obtained from biopolymers such as polysaccharides, proteins, and lipids, combined with active compounds, can be an alternative approach. This article focused on recent studies that used alternative biodegradable polymeric matrices in conjunction with natural compounds with antioxidant/antimicrobial activity on chicken meat. Its impact on physicochemical, microbiological, and sensory characteristics was evident, as well as the effect on its shelf-life. In general, different combinations of active edible films or coatings had a positive effect on the chicken meat. Different studies reported that the main results were a decrease in microbial growth and pathogen survival, a slowdown in lipid oxidation evolution, and an improvement in sensory quality and shelf-life (an increase from 4 to 12 days).

## 1. Introduction

Chicken meat was the most produced meat worldwide in 2020, representing 35% of the global production, followed by 33% of pork and 20% of beef [1]. Despite that, chicken meat is highly perishable, and it has a limited shelf-life even in refrigerated storage. Spoilage and sensory changes caused by physical, chemical, and biological agents lead to meat and meat products being unacceptable for consumers, which also contributes to food waste [2,3]. Meat spoilage is mostly caused by microbial deterioration, lipid oxidation, and autolytic, enzymatic reactions [4,5]. Spoilage results in color and texture changes, and the formation of off-flavors, off-odors, and slime, among other undesired characteristics [2].

Food packaging plays an important role in food protection and shelf-life extension. The environmental concerns about the use of plastic and derivates lead to the need to search for new packaging solutions [6,7]. Non-degradable plastic accounts for 73% of litter in any aquatic habitat on a global scale [8], with about 4.8–12.7 million metric tons of plastic waste entering the oceans per year [9]. In recent years, studies have been directed toward the use of biopolymers, and to replace synthetic polymers due to their capacity for recycling and degradation [10,11]. The interest in the use of edible films and coatings has grown due to promising results in food preservation since they are biodegradable, biocompatible, recyclable, and of renewable origin [12,13,14]. The technology has been used in food preservation for centuries. The first record found was the preservation of citrus fruits by coating them with molten wax made in China in the 12th and 13th centuries (Anonymous, 1944) [15]. The technology was also used to preserve and increase meat products’ shelf-life in the 16th century (Contreras-Medellin and Labuza, 1981, in [16]). Later in the 19th century, the use of gelatin to preserve meat was patented in the United States [17].

Edible films and coatings can be produced from polysaccharides, proteins, lipids, or biocomposites [12,16,18]. It is also possible to incorporate functional agents in the packaging that enables the creation of active packaging, improving the product’s characteristics and consequently increasing its shelf-life [18,19]. They are designed to deliberately incorporate components that release substances to the packaged foods or the environment that surrounds them or that absorb substances from these foods/environments.

Antimicrobial and antioxidant activities on edible films and coatings are promising forms of active packaging. There are several groups of antimicrobial and/or antioxidant compounds capable of being incorporated into edible films. However, due to the potentially toxic effects of synthetic additives, there is a growing interest in their substitution for natural additives [20,21,22,23].

Natural antimicrobial compounds obtained from microorganisms, animals, and plants have been tested for their antimicrobial potential against pathogens and spoilage bacteria in various food products [24,25]. Antimicrobial agents can enhance food safety and shelf-life by inhibiting, reducing, or retarding the growth of spoilage or pathogenic microorganisms on food surfaces [26,27,28,29]. Bactericidal and fungicide capacity, stability at temperature and pH, activity at low concentrations, non-transmitting color and taste, and low toxicity are some of the criteria that should be considered when choosing an antimicrobial compound [22,23].

Natural antioxidant agents can be obtained from plant extracts, essential oils (EO) from herbs and spices, and a wide variety of polyphenolic concentrates from waste biosources [23,30]. They contain active compounds that prevent lipid oxidation, delay the development of off-flavors, and improve color stability [20,31,32]. Moreover, they also have good antimicrobial and antifungal properties [30,33].

This review discusses recent advances and new solutions in meat packaging, focusing on edible films and coatings with the incorporation of compounds with antimicrobial and antioxidant properties to improve chicken meat sensory characteristics, safety, and shelf-life.

## 2. Edible Films and Coatings Definition and Obtention

Edible films and coatings are defined as thin layers made of natural polymers, used for wrapping or coating foods, representing an important role in their conservation, distribution, and marketing [34,35,36].

Edible films and coatings have different applications and a wide range of properties that could help the food industry to enhance quality and shelf-life. The addition of flavors, aromas, antimicrobial agents, antioxidants, pigments, and vitamins can improve food quality and reduce many problems with a direct impact on shelf-life [37]. The extensive use of petroleum-derived polymers and consequent packaging wastes cause adverse effects on environmental pollution [38]. These concerns allow the approach and the intensification of studies to other more sustainable, biodegradable, and non-polluting alternatives, such as edible films and coatings based on biopolymers. The films can be produced by wet or dry methods [39,40]. By wet processing, films (dried over a surface) and coatings (dried over foods) can be obtained [39]. Wet methods consist in spreading and solvent evaporation or “casting.” Edible coatings are formed by dipping, spraying, panning, fluidized bed, and enrobing, depending on the characteristics of the foods [39,41,42].

The production of edible films by dry methods can be achieved through extrusion or thermopressing/thermoforming [12,39]. The thermopressing/thermoforming technique is normally used for the production of plastic utensils and also involves high temperatures and pressure [12]. The technology has been recently applied to the production of bioactive films [43,44].

### 2.1. Types and Materials

Edible films and coatings can be obtained from hydrocolloids, lipids, and their composites [12,16,18]. The use of this type of material can have several purposes, such as a barrier to moisture migration and gas flow, preservation of mechanical properties, and protection against microbial oxidation and deterioration, which can be increased by the addition of functional compounds [45,46]. Hydrocolloids usually provide better mechanical properties than lipids and hydrophobic substances [16]. Polysaccharide hydrocolloids are the most used components in the production of edible films and coatings [47]. However, these and other natural film-forming substances may need film additives such as plasticizers, crosslinking agents, emulsifiers, and reinforcements to improve or modify the basic functionality of the material film, such as mechanical resistance, water resistance, and elasticity [16,48]. Due to the limited function of a single-component edible film, composite films can be obtained by multiple biopolymers combined to create edible films with desirable properties [18,40]. Table 1 summarizes the film-forming materials of films and coatings.

Furthermore, active edible films can be created by the addition of functional compounds as anti-browning agents, colorants, flavors, nutrients, probiotics, nutraceuticals, antimicrobial and antioxidant compounds, etc. [60,61,62]. Table 2 summarizes the main functional and bioactive ingredients of active films and coatings.

### 2.2. Functions, Properties, and Applications

Films and coating obtained by biopolymers have several benefits due to their edible nature, availability, low cost, and biodegradability [67]. They are designed to deal with food’s susceptibility to physical/mechanical impacts, chemical reactions, and microbial development [68,69,70].

The main characteristics of edible films and coatings are related to (1) edibility and biodegradability; (2) physical and mechanical protection, pressure, vibrations, and other mechanical factors; (3) prevent and control of mass transfer or barrier functions (e.g., water vapor barrier, oxygen barrier, UV light barrier, organic vapor barrier, fat barrier); (4) active substance carriers and controlled release; (5) improvement of sensory properties; (6) shelf-life extension and safety enhancement; and (7) convenience and quality preservation [39,71,72].

### 2.3. Antimicrobial and Antioxidant Edible Films for Chicken Meat

Meat spoilage can be mainly caused by lipid oxidation, autolytic, enzymatic reactions, and microbial deterioration [4,5]. Several factors influence meat spoilage, such as storage temperature, presence of oxygen, moisture, light, endogenous enzymes, and microorganisms [2,3]. Alone or in combination, the action of these factors can cause changes in the color, odor, texture, and flavor of meat [2]. Compared to red meat, chicken meat has a shorter shelf-life mainly due to lipid oxidation and resulting off-flavors because of its higher content of unsaturated fatty acids [73]. Additionally, the original microbiota and meat processing conditions may contribute to the shorter shelf-life [73].

Edible films and coatings can present some benefits and economic impact on the meat industry. The prevention of moisture and weight loss, sensory changes in texture, flavor, odor, color, and dripping reduction can positively influence meat quality. Furthermore, the low oxygen permeability leads to the reduction of lipids oxidation, myoglobin oxidation, spoilage and pathogenic microorganisms, and partial inactivation of deteriorative proteolytic enzymes [74].

The addition of plant extracts and essential oils has gained prominence due to their antimicrobial and antioxidant potential. The use of antioxidant biocompounds in edible films and coatings formulations is mainly to prevent lipid oxidation, delay the development of off-flavors, and improve color stability [20]. Their activity has the ability to prevent chain reactions that initiate peroxidation, scavenging oxygen-reactive species, inhibit pro-oxidative enzymes, breaking auto-oxidative chain reactions, capturing O^2−^ radicals and preventing the development of peroxides, and chelate metal ions, preventing the generation of reactive species or lipid peroxides decomposition [20,75].

Antimicrobial compounds can be obtained from microorganisms, animals, and plants. The main bioactive compounds of vegetal origin with antimicrobial activity include phenolics, terpenes, aliphatic alcohols, aldehydes, acids, and isoflavonoids [21,76]. These compounds may have different mechanisms of action in microbial cells, such as changes in cell membrane permeability, cytoplasmic membrane disintegration, the release of cellular constituents, changes in phospholipid and fatty acid composition, changes in DNA and RNA synthesis, and destruction in protein translocation [74,77].

Table 3 presents studies with different types of edible films and coatings on chicken meat and the main results obtained in each study.

Overall, different combinations of edible films or coatings had a beneficial effect on chicken meat. According to the results presented in Table 3, this is mainly due to the control of microbiological and lipid oxidation development.

Typical levels encountered at the processing step for aerobic colony count are <10^5^ CFU/g, and for *E. coli*, <10^2^ CFU/g [91]. Spoilage can be detected when the microbial population reaches about 10^7^–10^8^/cm^2^ [92], which may result mainly in off-flavors, off-odors, and slime [2]. According to different authors, the combination of edible films/coatings with bioactive ingredients allowed to slow down the development of deteriorative and pathogenic microorganisms. It was possible to identify similarities in the behavior of spoilage indicator microorganisms. In general, the increase of counts over time is common among the developed works; however, it is also common the slowing down the development of counts compared to samples without the application of films, or with the application of films without the addition of antimicrobial compounds. Khan et al. [78] reported counts for aerobic mesophilic bacteria above 6 log CFU/g in broiler meat samples contained in plastic bags after 11 days, whereas samples packed in composite gelatin films with curcumin remained below 6 log CFU/g until 18 days.

*Pseudomonas* spp. are the most important group of microorganisms responsible for the spoilage of fresh meat when their counts reach 7–8 log [28]. Mehdizadeh and Langroodi [66] reported a 2.81 log to reduce *Pseudomonas* spp. compared to the control samples with the use of chitosan combined with propolis extract (1%) and *Zataria multiflora* Boiss EO (1%). In another study, Raeisi et al. [28] reported that samples coated with alginate + 5 mg/mL rosemary EO; alginate + 5 mg/mL cinnamon EO + 2000 IU/mL nisin; alginate + 5 mg/mL rosemary EO + 2000 IU/mL nisin; and alginate + 5 mg/mL cinnamon EO + 5 mg/mL rosemary EO never reached to 7 log CFU/g for *Pseudomonas* spp, throughout the storage period (15 days, 4 °C).

Coating and film laying has also been demonstrated to have a beneficial effect in the reduction of pathogenic microorganisms populations. Nouri Ala and Shahbazi [32] performed a study of chicken breast samples immersed in *Listeria monocytogenes* suspension and then coated in carboxymethyl cellulose (CMC) with the incorporation of *Ziziphora clinopodioides* EO (ZEO) and *Mentha spicata* EO (MEO). Significantly bacterial reductions were observed in groups with CMC + ZEO 0.5% + MEO 0.5% and CMC + ZEO 0.25% + MEO 0.5% on day 14 compared to day 0 at 4 °C by 2.44–2.62 and 2.91–3.03 log CFU/g, respectively. The same authors reported count reductions for *Staphylococcus aureus*, *Escherichia coli* O157:H7, *Salmonella* Typhimurium, and *Campylobacter jejuni*. In another study, Ca-alginate coatings combined with nisin, cinnamon EO, and rosemary EO delayed the development of *L. monocytogenes* in more than 2 log CFU/g in the last day of storage of chicken meat (15 days, 4 °C) [28].

The measurement of thiobarbituric acid reactive substance (TBARS), peroxide value (PV), and total volatile basic nitrogen (TVBN) has been widely used to estimate the extent of meat deterioration. Several authors have reported lower values in TBARS, PV, and TVBN values in samples treated with films/coatings compared with samples without any treatment [29,32,65,66,80,83,89,90]. The presence of antioxidants and very low permeability to oxygen and carbon dioxide are justified as the main reasons for these results, according to the authors.

There are no reference values for thresholds TBARS values because the numbers are influenced by some variables such as animal species, dietary status, age of the animals, type of meat (raw or cooked), and types of TBA methods used [93]. Although, some authors used values between 1 and 2 mg MDA/kg as the maximum acceptable values for chicken meat at which rancid off-flavors become noticeable [29,53,89]. Zhou et al. [90] refer that TBARS values of more than 0.5 mg/kg in chicken meat may be detectable by consumers as off-flavor. All studies for TBARS, represented in Table 3, showed lower values for samples with films incorporated with antioxidant compounds compared without any treatment, and almost all studies showed a similar increasing trend during the time for all treatments applied. Dalvandi et al. [89] reported values between 0.29 and 1.21 mg MDA/kg in chicken breast meat for 16 days at 4 °C. In another study, values for TBARS of chicken samples at 4 °C were between 0.207 mg/kg and 1.823 mg/kg. The highest value was obtained for control samples on day 10. For films with konjac glucomannan and Kappa-carrageenan incorporated with camellia oil, TBARS values were always lower than 1 mg/kg [90].

Different thresholds for TVBN are used in the scientific community in different studies. Yousefi et al. [83] used a threshold of 15 mg/100 g. Mehdizadeh and Langroodi [66] used a threshold of 28 mg/100 g. Nouri Ala and Shahbazi [32] and Dalvandi et al. [89] used 25 mg/100 g as a threshold that assures meat product freshness. Nouri Ala and Shahbazi [32] reported values always below the threshold established in samples coated with carboxymethylcellulose incorporated with *Mentha spicata* and *Ziziphora clinopodioides* during 14 days at 4 °C. Bacterial spoilage may increase TVBN values; thus, microbial control through the application of films or coatings with active compounds may contribute to a substantial reduction of TVBN [89,90].

Other indicators, such as a decrease in pH changes, a reduction in cooking losses, and a decrease in weight and moisture losses in samples treated with films or coatings incorporated with natural extracts or EO, are also reported by several authors [29,80,83,85]. The use of films or coatings can delay moisture and weight loss due to the barrier effect, such as low moisture absorption and water vapor permeability [19]. However, this is only possible until the film/coating is saturated [86].

It is known that the pH of meat affects its microbial stability and shelf-life. Some studies with the application of chitosan have demonstrated the ability of pH stability over the total storage time. The authors mention that this result may be due to the use of acetic acid in the preparation of the solution [87,88]. By regulating and stabilizing the pH, edible films and coatings can help maintain the desired color of meat. Color is one of the main attributes to determine food acceptance by consumers. The color properties of meat packed with films or coated depend on the film-forming materials, but mainly on the concentration of the natural compounds added. The addition of compounds that influences the color can have a negative effect on the samples. However, over storage time, this impact may be lower than the impact of color changes in the untreated samples, as their deterioration due to lipid oxidation, pigment degradation, and microbial growth provides very pronounced color changes, unlike samples where films or coating with natural compounds are applied as demonstrated by Mehdizadeh and Langroodi [66]. Additionally, the barrier properties of edible films and coatings mentioned before can prevent the escape of juices and minimize the contact of meat with oxygen. This can help preserve the natural color of fresh meat, limiting oxidation and maintaining its desirable appearance.

Whenever sensory analysis was carried out, the application of films or coatings showed an improvement in the product, namely in the color stability, taste, texture, odor, and total acceptance of the product [31,32,66,83,85,87,88]. However, some authors reported that the increase of EO or extract concentrations decreased sensory ratings. According to Mahdavi et al. [29], the use of chitosan and anise EO at 1% did not change the chicken burgers’ odor, but sensory ratings decreased with the increase of EO concentrations. Mehdizadeh and Langroodi [66] reported that concentrations higher than 1% of propolis extract and *Zataria multiflora* Boiss EO affects the taste and reduces sensory ratings of chicken breast fillets. Thus, the choice of film-forming materials and their concentrations, especially antimicrobial/antioxidant compounds, is very important to avoid negatively influencing the meat’s organoleptic characteristics.

As a consequence of the reported results of several studies, an increase in shelf-life is also expected. Several studies have demonstrated an increase in the shelf-life of chicken meat with different combinations of films or coatings with the addition of antioxidant and antimicrobial compounds. Garavito et al. [86] prepared edible coatings from guar gum, nisin, and oregano EO and conducted preservation tests of chicken breast fillets. According to them, the application of the coating increased the product shelf-life to 9 days compared to the control samples (6 days). Khan et al. [78], in a study with films made of bovine gelatin + carrageenan and curcumin, reported a shelf-life increase of up to 17 days in fresh broiler meat compared to control samples (10 days). Raeisi et al. [28] reported an extended shelf-life of about 6 days in chicken breast meat coated with alginate solution containing 5 mg/mL of cinnamon EO and 5 mg/mL rosemary EO. Mehdizadeh and Langroodi [66] reported an extension of the shelf-life of chicken breast meat to approximately 16 days with the use of chitosan, propolis extract (1%), and *Zataria multiflora* Boiss EO (1%). Considering the microbiological results, and comparing them with the limits considered acceptable (6–7 log CFU/g) mentioned in these studies, it was possible to increase meat’s shelf life between 4 and 12 days. In some of them, the shelf-life was longer than 15 days [28,66,78,80,83,89]. These results are promising, since the shelf-life of chicken meat is about 4–5 days. However, the other parameters should also be taken into consideration, especially the sensory evaluation. Although some authors report acceptable results for sensory evaluation throughout storage, others do not present data. Despite the observed results pointing to an increase in the shelf-life, there are differences among them. These differences are probably due to the antimicrobial and antioxidant capacity of the different active compounds used, as well as the ability of the film-forming materials to maintain their integrity throughout the storage time.

The enhanced moisture retention, reduced microbial growth, and improved barrier properties provided by edible films can contribute to a longer shelf-life, allowing for a longer period of safe consumption. However, some disadvantages must be overcome. It is important to carefully consider the specific application and formulation of edible films to minimize any potential disadvantages and ensure their compatibility with chicken meat. Sensory changes due to film or coating composition may affect the appearance, texture, and taste of the meat. The adhesion and uniform coverage on the chicken surface can also be challenging. The quality and stability of edible films can be affected by environmental factors such as temperature, humidity, and light. Changes in these conditions during storage can lead to film degradation, loss of barrier properties, or decreased effectiveness over time. Despite these considerations, edible films and coatings remain excellent solutions for the future and should continue to be studied.

## 3. Conclusions

Environmental concerns demand the search for different approaches to food packaging. Edible films or coatings are a promising emerging packaging system as an alternative to the existing conventional petroleum-based systems. They can be made from biodegradable polymers and combined with functional compounds that can also be obtained from agroindustry waste which contributes to the circular economy and relieve environmental problems.

The use of edible films and coatings with antimicrobial/antioxidant properties on chicken meat has shown promising results. Based on recent studies, edible films and coatings can reduce the weight loss of chicken meat, thereby improving its overall sensory attributes and increasing consumer acceptability. Additionally, the incorporation of various antimicrobial agents, such as essential oils, bacteriocins, and plant extracts, helps control microbial growth and extend chicken meat’s shelf-life up to 12 days, according to recent studies. Furthermore, antimicrobial activity against common foodborne pathogens has been demonstrated, providing an additional layer of protection.

The results also showed improved oxidative stability of chicken meat with films or coatings with active compounds compared to untreated samples. The reduction in lipid oxidation helps maintain the overall quality and sensory attributes of the chicken meat, including its color, texture, and flavor.

Despite the promising results, further research is needed to optimize the formulation and application of edible films on chicken meat to overcome some obstacles related to film integrity and adhesion, as well as the product’s sensory characteristics.

## Figures and Tables

**Table 1:** Examples of film-forming materials of active films and coatings.

Types	Sources	Fil-Forming Materials
Polysaccharides	Seaweed Products	Alginate, carrageenans, agar.
[31,47,49,50]	Gums	Gum arabic, guar gum, basil seed gum, galbanum gum.
	Cellulose Derivatives	Methylcellulose, carboxymethyl cellulose, hydroxypropyl cellulose, hydroxypropyl methylcellulose.
	Others	Pectin, starch, chitosan, pullulan, konjac glucomannan.
Proteins [49,51,52,53]	Plant-derived	Corn zein, kafirin, wheat gluten, soy mung bean, pea, grass pea, wild and Pasankalla quinoa, bitter vetch.
	Animal-derived	Collagen, gelatin, caseins, whey, egg white, myofibrillar protein, keratin, surimi.
Lipids [49,54,55]	Oil, Fat, Shortening, Margarine	Animal and vegetable native oil and fats: peanut, corn, olive, sunflower, rapeseed, coconut, palm, palm kernel oil, cocoa, milk butter, lard, tallow, etc. Fractionated, concentrated, or reconstituted oils and fats: fatty acids, mono, di, and triglycerides, cocoa butter substitute, etc. Hydrogenated or trans-esterified oil: margarine, shortening, etc.
	Waxes	Natural vegetable waxes: candelilla, carnauba, jojoba, sugar cane, rice bran. Natural animal waxes: bees, whales, lanolin, insects, spermaceti. Nonnatural waxes: paraffin, mineral, microcrystalline, oxidized, or non-oxidized polyethylene.
	Natural Resins	Asafoetida, benjoin, chicle, guarana, myrrh, olibanum (incense), opoponax, sandarac, styrax resins.
Plasticizers	Monosaccharides	Glucose, mannose, fructose.
[56]	Disaccharides	Sucrose.
	Oligosaccharides	
	Polyols	Sorbitol, glycerol, mannitol, xylitol, glycerol derivatives and polyethylene glycols.
	Lipids and Derivatives	Phospholipids, fatty acids, surfactants, etc.
Crosslinking Agents [57]	Chemical Crosslinking	Disulfide bonds, aldehydes (formaldehyde, glutaraldehyde, cinnamaldehyde), oxidized polysaccharides, phenolic compounds in covalent crosslinking, TGase, alginic acid, Di- or polycarboxylic acids in covalent crosslinking (citric acid), genipin, condensation involving cystamine/cysteine, zero-length crosslinking induced by 1-methyl-3(3-dimethylaminopropyl)carbodiimide.
	Physical Crosslinking	Ionic crosslinking involving divalent cations, polyphenols with polysaccharides, polyelectrolyte complexes, di- or polycarboxylic acids in ionic crosslinking, TPP-chitosan ionic interactions.
Surfactants and Emulsifiers [58]		Glycerol monostearate, sucrose ester, sodium stearoyl lactate, sodium dodecyl sulfate, ethyl lauroyl arginate HCl, Span 20 to 80, Tween-20 to 80, soy lecithin.
Solvents [47,59]		Water, ethanol, acetic acid, water-ethanol mixtures.

**Table 2:** Examples of bioactive ingredients of active films and coatings [29,40,63,64,65,66].

Sources	Bioactive Ingredients
Essential oils (EO)	Basil, cinnamon, clove, garlic, ginger, lemon, oregano, anise, Zataria multiflora Boiss.
Plant extracts	Cinnamon, clove, garlic, grape seed, green tea, pomegranate, rosemary, sage, thyme, peanut skin extract, pink pepper residue extract, propolis.
Phenolic compounds	Tannins, flavonoids, simple phenols, phenolic acids, volatile phenols.
Enzymes	Lysozyme, lactoperoxidase.
Bacteriocins	Nisin, natamycin
Vitamins	Ascorbic acid, α-tocopherol
Natural pigments	Anthocyanin, β-carotene, curcumin

**Table 3:** Recent studies in edible films and coatings with antimicrobial/antioxidant properties on chicken meat.

Film-Forming Base Materials	Bioactive Ingredients	Process	Meat and Preservation	Main Results	Ref.
Bovine gelatin+ carrageenan	Curcumin; Gallic acid; Quercetin	Casting	Broiler meat (4 °C/18 days)	↓ Microbial growth: AMB; ↑ Shelf-life	[78]
Carboxymethyl cellulose+ Cellulose nanofiber	Inulin	Casting	Chicken fillets (4 °C/8 days)	↓ Microbial growth: AMB, TPB, TC; ↑ Shelf-life	[79]
Chitosan	Peanut skin EXT; Pink pepper residue *EXT*	Casting	Ground chicken meat (3 °C/7 days)	↓ Microbial growth: AMB, TPB; ↓ TBARS and PV increase; ↔ pH; ↑ Shelf-life	[65]
Chitosan	Grape seed EXT	Casting	Chicken breast fillets (4 °C/15 days, vacuum)	↓ Microbial growth: AMB, TC; ↔ pH; ↓TBARS increase; ↑ Shelf-life	[80]
Chitosan	Anise EO	Casting	Chicken burger (4 °C/12 days)	↓ Microbial growth: AMB, TPB, *Pseudomonas aeruginosa*, *Staphylococcus aureus*, *Escherichia coli* O157:H7; ↓ Moisture decrease; ↓ TBARS increase; ↑ Shelf-life	[29]
Chitosan nanofibers+ Bacterial cellulose nanofibers+ Bovine gelatin	*Lactobacillus casei*; *Bacillus coagulans*	Casting	Chicken breast fillets (4 °C/14 days)	↓ Microbial growth: AMB, TPB, LAB; *Enterobacteriaceae*, *Pseudomonas* spp.; ↓ Survival of pathogens: *Listeria monocytogenes*; ↓ pH increase; ↓ PV and TVBN increase; ↑ Sensory quality; ↑ Shelf-life	[7]
Kafirin	Citral; Quercetin	Casting	Chicken fillets (2 °C/4 days)	↓ Microbial growth: AMB; ↓ TBARS increase (except for citral) ↑ Shelf-life	[53]
K-carrageenan	Fenugreek seeds EXT	Casting	Chicken breast fillets (5 °C/7 days)	↓ Microbial growth: AMB; ↑ Shelf-life	[81]
Starch	Torch ginger EO	Casting	(3 °C/6 days)	↓ Microbial growth: TC; ↓ Weight loss; ↓ pH; ↓ TBARS increase; ↔ Sensory quality	[19]
Seed gum (*Alyssum homolocarpum*)	*Echinacea purpurea* (L.) EXT	Casting	Chicken meat (fridge temperature/14 days)	↓ Microbial growth: AMB; TC; *S. aureus*; ↓ pH increase; ↓ TBARS increase; ↑ Sensory quality	[82]
Ca-Alginate	Nisin; Cinnamon EO; Rosemary EO	Coating	Chicken meat (4 °C/15 days)	↓ Microbial growth: AMB, TPB, LAB, MY, *Enterobacteriaceae*, *Pseudomonas* spp.; ↓ Survival of pathogens: *L. monocytogenes*; ↑ Shelf-life	[28]
Ca-Alginate	Lactoperoxidase	Coating	Chicken breast (4 °C/16 days)	↓ Microbial growth: AMB, *Enterobacteriaceae*, *P. aeruginosa*; ↓ TVBN increase; ↔ TBARS and PV; ↓ pH changes; ↑ Sensory quality; ↑ Shelf-life	[83]
Ca-Alginate	Quercetin;	Coating	(6 °C/11 days)	↓ Microbial growth: AMB, TPB, *Enterobacteriaceae*, *Pseudomonas* spp.; ↓ pH increase; ↓ TVBN increase; ↑ Sensory quality	[84]
Ca-Alginate + whey protein	Lactoperoxidase	Coating	Chicken thigh (4 °C/8 days)	↓ Microbial growth: AMB, *Enterobacteriaceae*; ↑ Shelf-life	[64]
Sodium caseinate	Ginger EO	Coating	Chicken breast fillets (4 °C/12 days)	↓ Microbial growth: TPB, MY; ↔ TBARS; ↓ Cooking loss; ↑ Sensory quality; ↑ Shelf-life	[85]
Sodium Alginate; Galbanum Leo-resin gum	Ziziphora persica EO	Coating	Chicken fillets (4 °C/12 days)	↓ Microbial growth: AMB, TPB, LAB, MY, *Enterobacteriaceae*, *Pseudomonas* spp.; ↓ Survival of pathogens: *L. monocytogenes*; ↓ TBARS, TVBN, and PV increase; ↑ Sensory quality; ↑ Shelf-life	[31]
Guar gum	Nisin; Oregano EO	Coating	Chicken breast fillets (4 °C/16 days)	↓ Microbial growth: *Pseudomonas* spp.; ↑ Sensorial quality; ↓ Weight loss; ↓ pH changes; ↑ Shelf-life	[86]
Chitosan	Tomato plant EXT	Coating	Chicken fillets (4 °C/16 days)	↓ Microbial growth: AMB, TPB, TC; ↓ pH changes; ↑ Sensory quality; ↑ Shelf-life	[87]
Chitosan	Propolis EXT; Zataria multiflora Boiss EO	Coating	Chicken breast fillets (4 °C/16 days)	↓ Microbial growth: AMB, TPB, LAB; *Pseudomonas* spp.; ↓ TBARS and TVBN increase; ↓ pH increase; ↑ Sensory quality; ↑ Shelf-life	[66]
Chitosan	Grape seed EXT	Coating	Chicken breast (4 °C/21 days)	↓ Microbial growth: AMB, TPB ↓ TBARS increase; ↓ pH changes; ↑ Sensory quality; ↑ Shelf-life	[88]
Carboxymethyl cellulose	Ziziphora clinopodioides EO; Mentha spicata EO	Coating	Chicken breast fillets (4 °C/14 days)	↓ Microbial growth: AMB, TPB, *Pseudomonas* spp., *P. fluorescens*, *Enterobacteriaceae*; ↓ Survival of pathogens: *L. monocytogenes*, *S. aureus*, *E. coli* O157:H7, *Salmonella* Typhimurium, *Campylobacter jejuni*; ↓ TVBN and PV increase; ↑ Sensory quality; ↑ Shelf-life	[32]
Carboxymethyl cellulose	Black pepper seed EXT; Turmeric EXT	Coating	Chicken breast fillets (4 °C/16 days)	↓ Microbial growth: AMB, TPB; ↓ TBARS, TVBN, and PV increase; ↑ Sensory quality ↑ Shelf-life	[89]
Konjac glucomannan + Carrageenan	Camellia EO	Coating	Chicken meat (4 °C/10 days)	↓ Microbial growth: AMB, TPB, LAB; ↓ Weight loss; ↓ pH increase; ↓ TBARS and TVBN increase; ↑ Sensory quality; ↑ Shelf-life	[90]
Maize starch + sodium trimetaphosphate	Grape juice	Coating	Chicken breast (4 °C/8 days)	↓ Microbial growth: AMB, TPB, *Enterobacteriaceae*; ↓ pH increase; ↓ TBARS increase	[14]

Ref.—References; ↑—Increase; ↓—Decrease; ↔—Without changes; TBARS—Thiobarbituric acid reactive substance; TVBN—Total volatile basic nitrogen; PV—Peroxide value; EXT—Extract; EO—Essential oil; TPB—Total psychrotrophic bacteria; AMB—Aerobic mesophilic bacteria; MY—Molds and yeasts; TC—Total coliform; LAB—Lactic acid bacteria.

## Data Availability

No new data were created.
